# Nephroprotective Role of Zhibai Dihuang Wan in Aristolochic Acid-Intoxicated Zebrafish

**DOI:** 10.1155/2020/5204348

**Published:** 2020-12-02

**Authors:** Ping-Hsun Lu, Hsun-Yao Lee, Yan-Liang Liou, Sheng-Fen Tung, Ko-Li Kuo, Yau-Hung Chen

**Affiliations:** ^1^Department of Chinese Medicine, Taipei Tzu Chi Hospital, Buddhist Tzu Chi Medical Foundation, No. 289, Jianguo Rd., Shin-Tien, New Taipei City, Taiwan; ^2^Department of Chemistry, Tamkang University, No. 151, Ying-Chuan Road, Tamsui, New Taipei City 251, Taiwan; ^3^Division of Nephrology, Taipei Tzu Chi Hospital, Buddhist Tzu Chi Medical Foundation, No. 289, Jianguo Rd., Shin-Tien, New Taipei City, Taiwan; ^4^School of Medicine, Buddhist Tzu Chi University, No. 701, Zhongyang Road, Section 3, Hualien, Taiwan

## Abstract

Zhibai Dihuang Wan (ZDW) is an eight-herbal formula of traditional Chinese medicine. Clinically, it regulated immune activity and was used to treat diabetes and renal disease. In this study, we aimed to explore the nephroprotective effect of ZDW in an aristolochic acid- (AA-) intoxicated zebrafish model. We used a green fluorescent kidney transgenic zebrafish to evaluate the nephroprotective effects of ZDW by recording subtle changes in the kidney. Our results demonstrated that ZDW treatment can attenuate AA-induced kidney malformations (60% for AA-treated, 47% for pretreatment with ZDW, and 17% for cotreatment ZDW with AA, *n* = 50). Furthermore, we found that the expression levels of *tnfα* and *mpo* were decreased either in pretreatment or cotreatment groups. In conclusion, our findings revealed that AA-induced nephrotoxicities can be attenuated by ZDW. Therefore, we believe that zebrafish represent an efficient model for screening AA-protective Chinese medicine.

## 1. Introduction

Zhibai Dihuang Wan (ZDW) is an eight-herbal formula of traditional Chinese medicine used to enrich kidney function. Clinically, it was used to treat chronic kidney disease and diabetic nephropathy and to regulate immunity, even was used to treat chronic renal insufficiency and relieve the symptoms [1–4]. The laboratory results showed that ZDW is able to regulate the inflammatory response and heart function and to treat diabetic nephropathy [5, 6]. In rats, ZDW treatment could lead to protein alteration in immune response, especially in coagulation and complement cascades [6–8]. In addition, ZDW was shown to have a protective effect on gentamycin-induced apoptotic injury in renal tubular cells [8]. These studies highlight the importance of ZDW in clinical and biomedical uses.

Aristolochic acid (AA) is a natural compound which was found in some certain species of plants (*Aristolochia* or *Asarum Aristolochia*) and has been demonstrated to have some adverse effects in humans and other animals [9, 10]. These adverse effects include the following: gene mutation, kidney inflammation, tubule necrosis, and anemia [11, 12]. Basically, AA-containing products are not allowed to be used in humans. However, humans might be exposed to AA under some situations, such as misused AA-containing products or accidentally drank AA-contaminated water. For these reasons, it would be beneficial if some AA-preventative chemicals and/or natural products could be found.

Zebrafish (*Danio rerio*) has been described as a reliable model organism for toxicity study [13, 14]. In special, the transgenic zebrafish line Tg(*wt1b:EGFP*), in which the glomerulus (gl) and pronephric tubes (pt) were labeled by green fluorescent protein (GFP), has been effectively used in many toxicological studies [15]. In the present study, we performed a series of time- and dose-dependent exposure experiments to assess the nephroprotective effects of ZDW in the transgenic line Tg(*wt1b:EGFP*). Subtle changes in pt and gl were easily observed by the detection of GFP signals or by staining with a well-known renal tubular differentiation marker, Na^+^/K^+^-ATPase *α*1 subunit (*α6F*) [16]. The protective effects of ZDW would be evaluated in this current study.

## 2. Materials and Methods

### 2.1. Fish Embryo Maintenance and Staging

Mature zebrafish of the wild-type (WT; AB strain) and Tg(*wt1b:EGFP*) strains [15] were maintained at the zebrafish facility of Tamkang University. AB strain zebrafish were purchased from Taiwan Zebrafish Core Facility (Academia Sinica, Taipei, Taiwan). Tg(*wt1b:EGFP*) stain was provided by Dr. Englert (Fritz Lipmann Institute, Germany) [15]. Fish maintenance, embryo collection, and embryonic stage identification are according to the standard procedures as described before [13]. Once the embryos were collected, they were divided into the test groups for the subsequent ZDW treatment experiments. All animal experiments in this study were performed in accordance with the guidelines issued by the animal ethics committee of Tamkang University.

### 2.2. Drug Treatment

Aristolochic acids (AA, CH_3_CONHC_6_H_4_OH; Sigma) were purchased from Sigma Pharmaceutical Co. (USA). The powders of ZDW were purchased from SunTen Pharmaceutical Co. (Taipei, Taiwan). ZDW are composed of 8 different herbs: Zhi Mu (*Rhizoma Anemarrhenae*), Huang Bai (*Cortex Phellodendri*), Shu Di Huang (*Radix Rehmanniae Preparata*), Shan Zhu Yu (*Fructus Corni*), Shan Yao (*Rhizoma Dioscoreae*), Fu Ling (*Poria*), Mu Dan Pi (*Cx. Moutan*), and Ze Xie (*Rhizoma Alismatis*). The detailed protocol for the preparation of ZDW powder was described in the previous study [8]. In brief, the powder of ZDW (100 mg) was dissolved in 100 mL distilled water (1000 ppm) at 37°C for 1 h and centrifuged for 800 rpm for 10 min, and then, the supernatant was applied in the following experiments.

**Figure 1:**
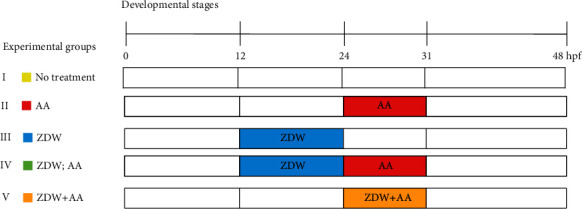
Schematic representation of experimental protocols performed in this study. Five groups (I-V) were applied in the treatment experiments based on combinations of different exposure onsets: (I) no treatment group, water only; (II) AA group, exposed to AA from 24 to 31 hpf; (III) ZDW group, exposed to ZDW from 12 to 24 hpf; (IV) ZDW, AA group (defined as pretreatment), exposed to ZDW from 12 to 24 hpf, withdrawn ZDW and followed by exposure with AA from 24 to 31 hpf; (V) ZDW+AA group (defined as cotreatment), coexposure with AA and ZDW from 24 to 31 hpf.

In zebrafish pronephros development, the specification of the mesodermal cells to the nephric lineage happens by 12 hpf, epithelialization of the duct appears at around 16-24 hpf, nephron patterning appears at around 30-40 hpf, and the final step, angiogenesis, appears at around 40-48 hpf [17]. According to these developmental stages, we used five exposure groups in this study to investigate the protective effects of ZDW, as shown in Figure 1. These exposure groups are as follows: (I) no treatment group, water only; (II) AA group, exposed to AA from 24 to 31 hpf; (III) ZDW group, exposed to ZDW from 12 to 24 hpf; (IV) ZDW; AA group (defined as pretreatment), exposed to ZDW from 12 to 24 hpf, withdrawn ZDW, and followed by exposure with AA from 24 to 31 hpf; (V) ZDW+AA group (defined as cotreatment), coexposed with AA and ZDW from 24 to 31 hpf.

For ZDW dose titration, AB strain embryos were collected, randomly divided into 50 per experimental group, and exposed to either water (no treatment, control: 0 ppm) or water containing ZDW (100, 500, and 1000 ppm) and/or 3 ppm AA. All embryos were cultivated in 6-well cell culture plates, and survival rates were determined at 48 hpf. For kidney malformation recording, the kidneys of drug-treated Tg(*wt1b:EGFP*) embryos at 48 hpf were compared with the kidneys of healthy embryos from the no treatment control group and subjectively classified as normal or malformed kidneys based on the observation of green fluorescence. The embryo displaying normal morphology in the regions of pt and gl was classified as the embryo with normal kidney, while the embryo which displayed defective morphology in either pt or gl was classified as the malformed kidney.

### 2.3. Histology, Immunohistochemistry, and Images

The procedures for embedding and cryosectioning were performed as previously described [17] except that embryos from all groups at 48 hpf were used and fixed in Dent's buffer (80% methanol, 20% DMSO). The localization of the pt in the cryosections was visualized by immunohistochemistry using a mouse monoclonal antibody (*α*6F, Developmental Studies Hybridoma Bank) and detected using an ABC staining system (Santa Cruz) and 3,3′-diaminobenzidine (DAB) as chromogens. Sections of 10 *μ*m in thickness obtained from the pronephric regions were subjected to toluidine blue stain for counterstaining. For images, all live or stained embryos were examined under a microscope (DM 2500, Leica) equipped with Nomarski differential interference contrast optics and a fluorescent module having a GFP filter cube (Kramer Scientific), and the images were captured with a Canon digital camera (Canon, Japan).

### 2.4. Quantitative Reverse Transcription-Polymerase Chain Reaction (qRT-PCR)

The experimental procedures of qRT-PCR have been previously described [10, 18, 19], except that primer sets were synthesized for detecting the expression levels of *cyclooxygenase 2* (*cox2*), *tumor necrosis factor α* (*tnfα*), and *myeloperoxidase* (*mpo*). The housekeeping gene *β-actin* was used as an internal control for normalization quantification.

## 3. Results

**Figure 2:**
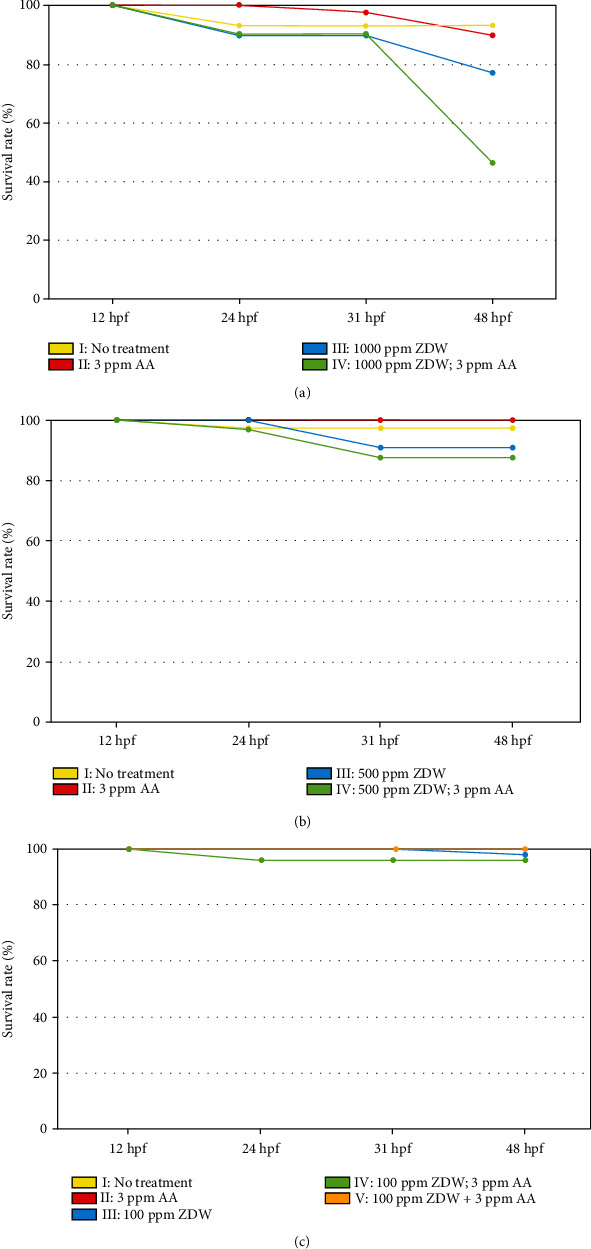
ZDW dose titration and survival rate analysis in this study. For ZDW dose titration, wild-type (WT; AB strain) embryos were collected, randomly divided into 50 per experimental group, and exposed to either water (no treatment, control: 0 ppm) or water containing ZDW (1000, 500, and 100 ppm, (a)–(c)) and/or 3 ppm AA. All embryos were cultivated in 6-well cell culture plates, and survival rates were determined at 12, 24, 31, and 48 hpf. The *x*- and *y*-axes represent the developmental stages and embryo survival rates, respectively. The cotreatment group (V) was only applied in low-dose ZDW (100 ppm).

For the titration of ZDW dose, zebrafish embryos were collected, randomly divided into several groups (50 embryos per group), exposed to the selected concentrations (0, 100, 500, and 1000 ppm), and determined the survival rates at 48 hpf. Our results showed that 91.9% to 100% (*n* = 50 (number of tested embryos in each group), *N* = 3 (triplicate experiments)) of the embryos were alive at 48 hpf after exposure to 100 and 500 ppm of ZDW. However, the survival rate decreased to 76.9% (*n* = 50, *N* = 3) when the exposure dose was increased to 1000 ppm (Figure 2). Thus, we proposed that exposure to 100 and 500 ppm of ZDW is unable to affect the embryos' survival and that these dosages may be well suitable for the subsequent toxicological evaluation.

**Figure 3:**
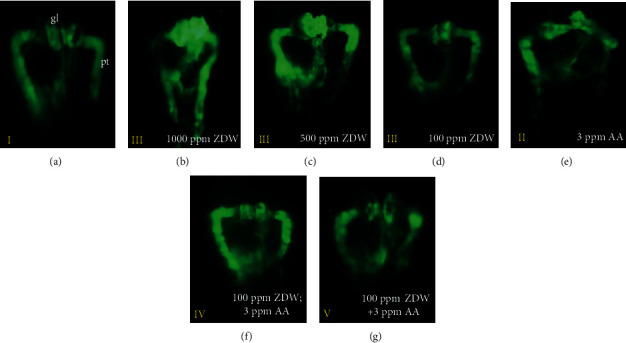
Kidney phenotypes of zebrafish embryos after prevention of ZDW. Morphological changes of Tg(*wt1b*:EGFP) embryos after exposure to water (a) or water containing 1000, 500, and 100 ppm of ZDW (b–d). AA-induced malformed kidney phenotypes which included curved pronephric tube (pt) and swollen, asymmetrical distribution of glomerulus (gl) were observed after being exposed with 3 ppm AA (e). Those phenotypic changes could be attenuated by either pretreatment (3 ppm AA; 100 ppm ZDW) (f) or cotreatment (3 ppm AA+100 ppm ZDW) with ZDW (g). Representative figures are shown here. All photos were taken from a dorsal view at the developmental stage of 48 hpf.

**Figure 4:**
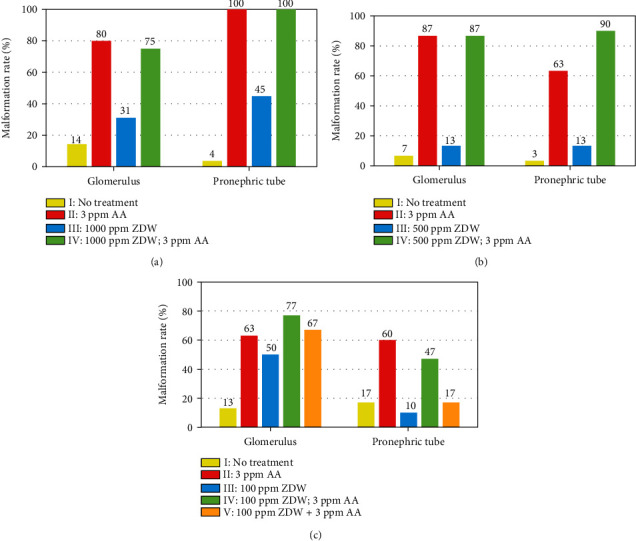
Malformation rates of kidney-defective phenotypes of zebrafish embryos after prevention of ZDW. Malformation rates of Tg(*wt1b*:EGFP) embryos after exposure to different doses of ZDW ((a): 1000 ppm; (b): 500 ppm; (c): 100 ppm) in combination with or without 3 ppm of AA were calculated. Malformation rates for each treatment group were calculated at 48 hpf. The cotreatment group (V) was only applied in low-dose ZDW (100 ppm).

We next assessed the potential teratogenic effect of ZDW on kidney development using the transgenic line Tg(*wt1b:EGFP*). The Tg(*wt1b:EGFP*) transgenic line, in which the gl and pt were labeled by GFP (Figure 3(a)), allows live imaging and timely observation of renal structure [15]. Using this transgenic zebrafish line, we conducted the abovementioned exposure protocols (Figure 1) to investigate the phenotypic defects caused by AA and/or ZDW. At 48 hpf, the embryos treated with either 3 ppm AA or 1000 ppm ZDW displayed malformed kidney phenotypes, such as (1) a separated and/or swollen gl and (2) a curved pt (Figures 3(b) and 3(e) vs. Figure 3(a)). In special, all AA-treated embryos displayed a curved pt, whereas these phenotypic changes appeared in 45% of embryos after 1000 ppm ZDW treatment (Figure 4(a)). At lower doses of ZDW exposure (500 and 100 ppm), fewer phenotypic defects in the pt (13% and 10% for 500 and 100 ppm ZDW, respectively) were observed (Figures 3(c) and 3(d) vs. Figure 3(a); Figures 4(b) and 4(c)). These observations suggested that 1000 ppm of ZDW treatment led to a higher kidney malformation rate (45%). In this regard, we chose 100 ppm of ZDW as the exposure concentration for the following experiments.

We further evaluated the protective effects of 100 ppm ZDW in the AA-exposed embryos. As shown in Figure 4(c), our results showed that in the pretreatment group (100 ppm ZDW; 3 ppm AA), ZDW had no obviously protective effects on zebrafish gl (63% vs. 77%). However, the malformation rates of pt decreased (60% vs. 47%). A similar scenario was observed in the cotreatment (100 ppm ZDW+3 ppm AA) group (malformation rate for gl: 63% vs. 67%; for pt: 60% vs. 17%). These observations suggested that 100 ppm ZDW treatment (in both pretreatment and cotreatment groups) had protective effects on zebrafish pt.

**Figure 5:**
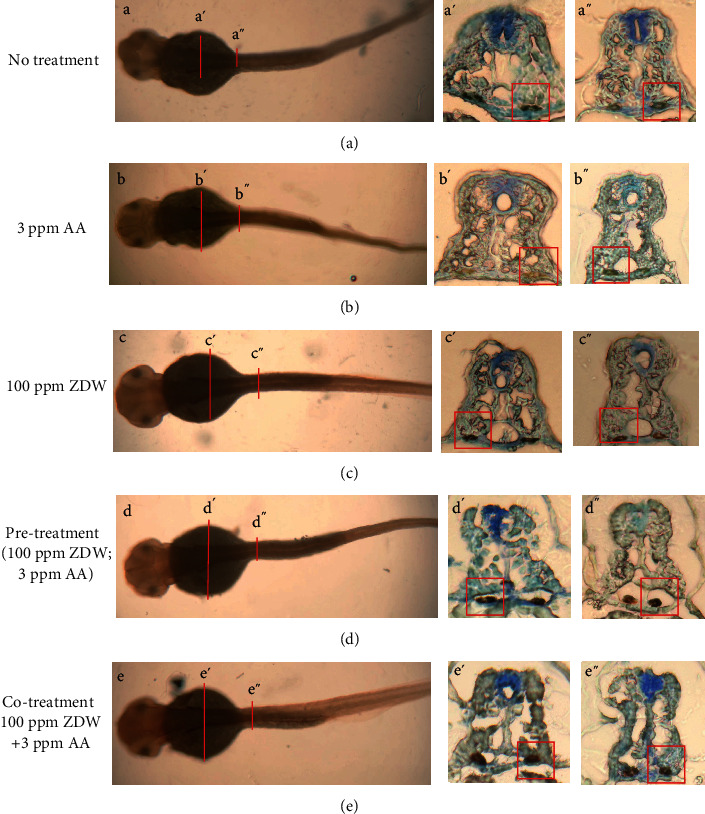
ZDW pretreatment attenuated AA-induced defective pronephric ducts. Immunohistochemical staining of the basolateral marker Na^+^/K^+^-ATPase alpha1 subunit (*α*6F) was performed on transverse sections of the pronephric tubes from 48 hpf zebrafish embryos after exposure to water (no treatment group, (a)), 3 ppm of AA (b), 10 ppm of ZDW (c), and AA combined with ZDW (pretreatment (ZDW; AA group) (d) or cotreatment (ZDW+AA) (e)). The sections were stained with a monoclonal antibody (*α*6F) and counterstained with hematoxylin. (a′)-(a^″^), (b′)-(b^″^), (c′)-(c^″^), (d′)-(d^″^), and (e′)-(e^″^) were different positions of the embryos indicated by red lines shown in (a), (b), (c), (d), and (e), respectively.

We intended to examine the defective renal phenotypes from each group at the histological level. The epithelial cells of pt in the embryos derived from each group were visualized by the *α*6F monoclonal antibody. The pt either in the no treatment control embryo or in the 100 ppm ZDW-treated embryo were a compact structure composing of a single layer of epithelial cells (Figures 5(a), 5(a′), 5(a^″^), 5(c), 5(c′), and 5(c^″^)). The 3 ppm AA-treated embryos displayed disorganized and broken epithelial cells around the pt regions (Figures 5(b), 5(b′), and 5(b^″^)). Interestingly, embryos derived from both pretreatment (100 ppm ZDW; 3 ppm AA) and cotreatment (100 ppm ZDW+3 ppm AA) groups displayed a more compact and more regular structure of the pt epithelial cells (Figures 5(d), 5(d′), 5(d^″^), 5(e), 5(e′), and 5(e^″^)). The above finding further strengthened that ZDW pretreatment or cotreatment ameliorates AA-induced defective renal phenotypes.

**Figure 6:**
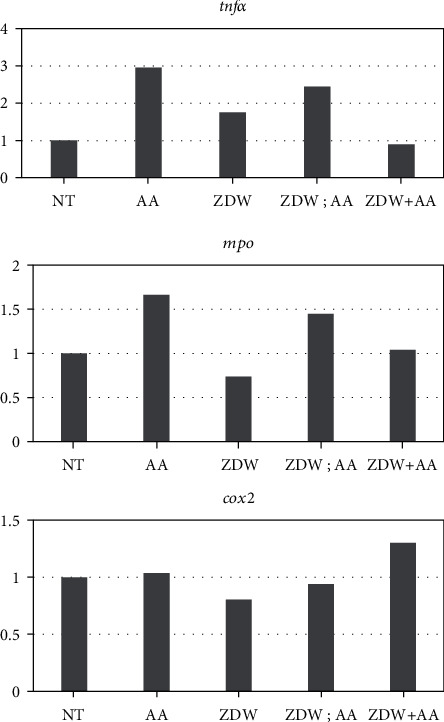
Quantitative gene expression was analyzed by quantitative reverse transcription-polymerase chain reaction (qRT-PCR). The total RNA was extracted at the 48 hpf, and the relative expression levels of the indicated genes were determined by qRT-PCR. The gene expression results of the indicated genes between no treatment control (NT) and different experimental groups. The data in triplicate were normalized for zebrafish *β*-actin expression.

Our previous study showed that AA treatment caused kidney malformations in zebrafish, and the expression of proinflammatory genes *tnfα* and *mpo* in the AA-treated embryos was also significantly increased [17]. To further investigate the molecular mechanisms of ZDW which ameliorate AA-induced defective renal phenotypes, the expression of proinflammatory genes (*cox2*, *mpo*, and *tnfα*) was determined by RT-qPCR. As shown in Figure 6, we found that the expression levels of *tnfα* and *mpo* in the AA-treated embryos were increased by 2.96 and 1.67 folds, respectively, compared with the no treatment control embryos (1 fold). In contrast, the expression levels of *tnfα* and *mpo* in the pretreatment embryos were decreased to 2.45 and 1.45 folds (0.9 and 1.04 folds in the cotreatment group), respectively, compared with the AA-treated embryos. However, ZDW treatment seems to have no obvious effects on the expressions of *cox2*. These results suggest that the attenuation of AA-induced renal malformation by either pretreatment or cotreatment of ZDW may be mediated by suppression of proinflammatory gene expression, such as *tnfα* and *mpo*.

## 4. Discussions

In this study, our findings reveal that AA-induced nephrotoxicity can be attenuated by cotreatment with ZDW. Previous studies have shown that ZDW possesses protective effects. For example, the study of the protective effect of ZDW on gentamycin-induced renal tubular cell apoptosis in mice suggests ZDW can reduce apoptosis and necrosis [8]. Another study showed ZDW can improve oral cell inflammation and the imbalance of metabolism by inhibiting NF-*κ*B and enhancing the activity of the ARE signaling pathway to ameliorate oxidative stress in the cultured cells [5]. Thus, we suggested the protective effect of ZDW is efficient in different experimental models.

It was shown that AA-induced nephrotoxicity is mainly due to inflammation [18, 19]. In this regard, whether ZDW exposure regulates the immune response and how many anti-inflammatory agents in the ZDW is an important issue that should be discussed. It has been demonstrated that L-selectin, Plg, and Kng1 (inflammatory markers) were upregulated after ZDW treatment, suggesting that ZDW may regulate inflammatory reaction [2]. In addition, previous research thought the anti-inflammatory effects of ZDW come from its antioxidant properties of five components: Fu Ling (*Poria cocos*), Zhi Mu (*Rhizoma Anemarrhenae*), Shu Di Huang (*Radix Rehmanniae Preparata*), Shan Zhu Yu (*Fructus Corni*), and Huang Bai (*Cortex Phellodendri*) [20–24]. For example, Fu Ling extract regulates the inflammatory response of macrophages via inhibition of iNOS, COX-2, IL-1*β*, and TNF-*α* through inactivation of the NF-*κ*B signaling pathway [24–26]; the aqueous extract of Zhi Mu and Shan Zhu Yu was reported to exert it has anti-inflammatory and analgesic effects by suppressing COX-2 and iNOS expression through the downregulation of NF-kappaB binding activity [27–31]. In addition, many animal studies reported that Fu Ling possesses protective effects against chemical-induced liver injuries [32, 33]; Shan Zhu Yu can be used to treat diabetic or epileptic rats through the regulation of their immune function [28, 34]. These observations supported our results, suggesting that the attenuation of AA-induced renal malformation by either pretreatment or cotreatment of ZDW would be mediated by the suppression of the proinflammatory gene expression (Figure 6).

In summary, these observations support the pharmacological basis of ZDW as a traditional herbal medicine for the treatment of inflammation and its associated disorders. In conclusion, this strategy and the zebrafish model provide an efficient method for studying the nephroprotective effects of ZDW during zebrafish development.

## Data Availability

All data used to support the findings of this study are available from the corresponding author upon request.
